# Effect of Hepatitis B Virus Genotypes on the Efficacy of Adefovir Dipivoxil Antiviral Therapy

**DOI:** 10.5812/hepatmon.10813

**Published:** 2014-08-17

**Authors:** Zhili Wen, Haiming Zhang, Meiying Zhang, Deming Tan, Qinghua Li, Hua Zhang, Ping Wu, Le Deng

**Affiliations:** 1 Department of Hepatology, Infectious Disease Hospital of Nanchang University, Jiangxi, China; 2 Department of Medicine, Hospital of Nanchang University, Nanchang, Jiangxi, China; 3 Department of Infectious Disease, Xiangya Hospital of Central South University, Changsha, Hunan, China; 4 Department of Medicine, People’s Liberation Army Hospital 163, Changsha, Hunan, China

**Keywords:** Hepatitis B virus, Genotype, Adefovir Dipivoxil

## Abstract

**Background:**

Hepatitis B is a common infectious disease in China. Many studies have shown that the genotype of hepatitis B virus (HBV) is probably associated with the efficacy of some antiviral drugs such as interferon α (IFN-α) and Lamivudine (LAM). However, the association between HBV genotype and adefovir dipivoxil (ADV) is controversial. ADV is the most popular antiviral drug in China due to its low price, good antiviral efficacy, few side effects, and convenient of administration.

**Objectives:**

This study focused on the effect of HBV genotypes on antiviral efficacy of ADV in patients with chronic hepatitis B infection (CHB).

**Patients and Methods:**

A total of 526 HBeAg-positive patients with CHB were randomly allocated into two groups. One group took ADV and another group took placebo. Nested Polymerase Chain Reaction (PCR) with multiple pairs of genotype-specific primers (nPCR-MPP) was used to analyze genotypes of HBV in these patients. Antiviral efficacy after treatment for three, six, 12 months was compared among the patients with different HBV genotypes.

**Results:**

Genotype B (73.6%) and genotype C (26.4%) were detected in these patients. After treatment for 12 months, the rate of HBV DNA seroclearance, ALT normalization, and HBeAg seroconversion were significantly higher in ADV group than in placebo group (P < 0.05). However, there were no significant differences in these three rates between patients infected with genotype B and C (P > 0.05).

**Conclusions:**

HBV genotypes B and C have no significant difference in virologic, biochemical, and immunologic response to ADV.

## 1. Background

Numerous studies have shown that genotype of hepatitis B virus (HBV) is associated with the condition of patients with chronic hepatitis B (CHB). For example, the patients with HBV genotype B have higher HBeAg seroconversion rate and lower carcinogenesis rate than those with genotype C have (1). In addition, HBV genotype is probably associated with the antiviral efficacy. Antiviral drugs mainly include interferon α (IFN-α) and nucleoside/nucleotide analogues. It has been well known that HBeAg-positive patients with genotype B HBV have higher response rate to IFN-α than those with genotype C have, and HBeAg-positive patients with genotype A have higher response rate to IFN-α than those with genotype D have (2-4). Thus, patients with CHB who are infected with different genotypes should take different doses for different periods when they are treated with IFN. It is recommended to determine HBV genotype before treatment with IFN. Lamivudine (LAM) is the earliest used nucleoside analogue in the world and the association between HBV genotype and LAM is very clear. Two meta-analyses (5, 6) showed that genotype B has same response to LAM as genotype C. The results suggested that it is unnecessary to focus on HBV genotypes for patients who want to receive antiviral treatment with LAM. Adefovir dipivoxil (ADV) is the most popular nucleotide analogue in China due to its low price, good antiviral efficacy, few side effects, and convenient prescription. However, the association between HBV genotype and ADV is controversial. It is not clear whether ADV-treated patients should check their HBV genotype and whether they should change drug dose and treatment period according to HBV genotype. Moreover, prognosis for patients with CHB condition in future is very different. Therefore, it is very important to know whether HBV genotypes affect antiviral efficacy of ADV.

## 2. Objectives

We used nested Polymerase Chain Reaction (PCR) with multiple pairs of genotype-specific primers (nPCR-MPP) to determine the genotypes of HBV in patients with CHB who received antiviral therapy with ADV, and to compare antiviral efficacy among patients with CHB who were infected with different HBV genotypes in order to define the association between HBV genotype and ADV efficacy. Through exploring the effect of HBV genotypes on antiviral efficacy, we tried to help doctors to use ADV reasonably and assess the prognosis of patients’ condition.

## 3. Patients and Methods

### 3.1. Main Equipment and Reagents

The employed equipment and materials were as follows: DNA Thermal Cycler (PTC-100,Germany); ultraviolet spectrophotometer (UV-7, Heima Medical Apparatus Ltd., Zhuhai, China); electrophoresis meter with constant voltage and current (DF-D, oriental materials and scientific center, Beijing, China); automatic GIS gel image processing instrument (UV-2000, Tannon Ltd., Shanghai, China); Serum DNA Extraction Kit (Axygen, USA); Taq plus DNA polymerase, dNTP, 10× Taq plus Buffer, Marker (DGL2000), gelose (Dingguo Biological Ltd., Beijing, China); and primers (Sangon Biological Engineering Technology & Services Co., Ltd., Shanghai, China). Specific Primers including two outer-primers and eight inter-primers were designed according to Wen ZL (7) and Naito H (8) to conserved sequence of HBV pre-S gene and S gene. These primers were used for PCR and could amplify all of specific gene sequence of six HBV genotypes (genotype A through F), making the length of PCR products different for every HBV genotype.

### 3.2. Patients

A total of 526 HBeAg-positive patients with CHB were randomly selected from the outpatients and the inpatients in Department of Infectious Disease, affiliated hospital with Nanchang University (October 2009 to October 2011) and were randomly allocated into two groups. The patients consisted of 375 men and 151 women aged from 18 to 65 years old and all were born in Jiangxi Province of China. One group was comprised of 252 patients including 184 men and 68 women, with a mean age of 38.1 ± 12.5 years old. Another group was comprised of 274 patients including 191 men and 83 women, with a mean age of 36.5 ± 10.3 years old. Informed consent was obtained from each patient and the study protocol conformed to the ethical guidelines of the 1975 Declaration of Helsinki as reflected in a priori approval by the Institutional Human Research Committee. Randomization was performed through random digits table. All of the patients were diagnosed according to “Guideline for Prevention and Treatment of Chronic Hepatitis B”, which was enacted in 2005 by Chinese Society of Hepatology and Chinese Society of Infectious Diseases, Chinese Medical Association (9). HBsAg was positive for at least six months in these patients’ sera, with a HBV DNA level of 10^ 4^ to 10^ 7^ IU/mL and ALT level of 80 to 400 U/mL. These patients had not received antiviral therapy for preceding six months. The patients who were coinfected with HIV, HCV, and HDV, had positive results for autoantibody, or had decompensated hepatosis, hyperthyroidism, or psychosis as well as pregnant women were excluded. The patients in the first group took 10 mg of ADV (Pharmaceutical Ltd. of Tianjin Pharmaceutical Research Institute, Tianjin, China) orally once a day for 48 weeks (ADV group). The patients in the second group took placebo orally once a day for 48 weeks as a negative control (NC group or placebo group). All of the patients were followed up for at least one year after therapy. Their compliance and adverse reaction to ADV or placebo were recorded and their sera were collected to detect HBsAg/HBsAb, HBeAg/HBeAb, HBV DNA level, and liver function after treatment for three, six, and 12 months.

### 3.3. Extraction of Hepatitis B Virus DNA

HBV DNAs in patients’ blood were extracted using the AxyPrep Humoral Virus DNA Extraction Kit (Axygen, United States) in accordance with the manufacturer’s instructions.

### 3.4. Genotyping of Hepatitis B Virus

The genotypes of HBV were determined by nested PCR with multiple pairs of genotype-specific primers (7). The reaction system in the first run of PCR was 25 µL, including 1 μL of DNA template, 0.5 μL of Taq plus DNA polymerase (2 U/μL), 0.5 μL of dNTP (10 mM), 2.5 μL of 10× PCR buffer (containing 20-mM Mg^2+^), 0.5 μL of P 1 (10 pmol/μl), 0.5 μL of P 2 (10 pmol/μL), and double distilled water (DDW). The amplification condition was 94°C for ten minutes, thirty 60-second cycles at 94°C, 45 seconds at 55°C, 90 seconds at 72°C, and five minutes at 72°C. The product from the first reaction underwent a second run with eight genotype-specific inter-primers, including B2, BA1R, BB1R, BC1R, B2R, BD1, BE1, and BF1. The reaction system was the same as that in the first run of PCR except addition of DNA template and primers. The amplification condition was ten minutes at 95°C, forty 20-second cycles at 94°C, 20 seconds at 58°C, 30 seconds at 72°C, and ten minutes at 72°C. The products in the second run of PCR were added into 1.5% gelose to perform electrophoresis and were dyed by ethidium bromide. The DNA segments from HBV genotype A through F appeared in different length and were estimated according to Marker (DL2000), by which the genotypes of HBV was determined.

### 3.5. Statistical Analysis

For statistical analysis, SSPS 17.0 (SPSS Inc., Chicago, IL, USA) was used. Measurement data were expressed as mean ± SD and were analyzed by independent-samples t test. The quantitative data were expressed as percentage and were analyzed by Chi square test. Statistical significance was assessed by one-way ANOVA followed by Bonferroni/Dunn testing. P value < 0.05 was considered as statistical difference and P value < 0.01 was considered statistically significant difference.

## 4. Results

### 4.1. Genotyping of Hepatic B Virus by nPCR-MPP

**Figure 1.**
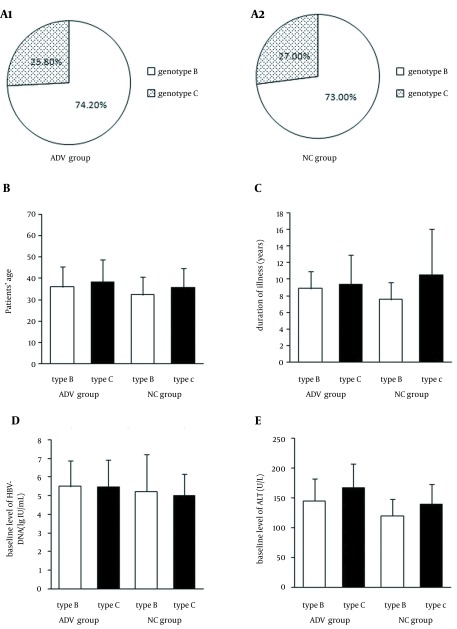
Genotypes B and C in Patients with Chronic Hepatitis B in Adefovir Dipivoxil Group and Control Group Before Treatment There were no significant difference in HBV genotype between ADV group and NC group before treatment (A1 and A2) (P > 0.05). There were also no significant difference in age, duration of illness, baseline level of HBV DNA, and ALT between ADV group and NC group, as well as between patients infected with genotypes B and C (B, C, D, E) (P > 0.05). All values were expressed as means ± SE.

The HBV genotype in all of the 526 patients with CHB was B or C. Not surprisingly, genotype A, D, E, and F as well as mixed genotypes were not found. Genotype B was more prevalent in the 526 patients with CHB than genotype C was. Totally, 387 patients (73.6%) had infection with genotype B and 137 (26.4%) with genotype C. Among 252 patients of ADV group, 187 patients (74.2%) had infection with genotype B and 65 (25.8%) with genotype C. In 200 patients of NC group, 274 patients (73.0%) had infection with genotype B and 74 (27.0%) with genotype C. There was no difference between ADV group and NC group in distribution of HBV genotype (P > 0.05). Before therapy, there were no significant difference between ADV group and NC group in sex, age, duration of illness, baseline level of HBV-DNA, and ALT (P > 0.05). Meanwhile, there were no significant difference between patients infected with genotype B and genotype C before therapy in sex, age, duration of illness, baseline level of HBV-DNA, and ALT (P > 0.05) (Figure 1).

### 4.2. Relation Between Hepatic B Virus Genotype and Antiviral Efficacy of Adefovir Dipivoxil

**Figure 2.**
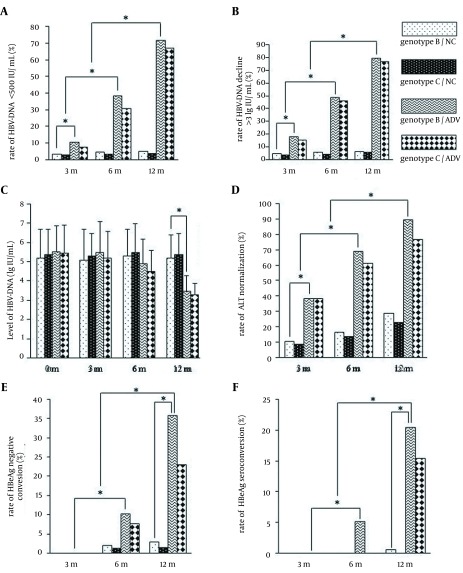
Association Between Hepatic B Virus Genotype and Antiviral Efficacy of Adefovir Dipivoxil To compare the response of HBV genotypes B and C to ADV, the HBV DNA seroclearance rate (< 500 IU/mL) (A), HBV DNA decline rate (> 3 lg IU/mL) (B), HBV DNA level (C), ALT normalization rate (D), HBeAg seroclearance rate (E), and the HBeAg seroconversion rate (F) in adefovir dipivoxil and placebo groups were assessed after treatment for three, six, and 12 months. *, P ≤ 0.05 was considered statistically significant. The values are expressed as means ± SE.

After three, six, and 12 months of treatment with ADV, the conditions of patients in ADV group improved significantly in comparison with the conditions of patients in NC group. There were significant difference between two groups in the incidence of HBV DNA seroclearance (HBV DNA level < 500 IU/mL), the incidence of HBV DNA decline (> 3 lg IU/mL), the level of HBV DNA, the incidences of ALT normalization, HBeAg seroclearance and HBeAg seroconversion (P < 0.05). However, there were no significant differences between patients infected with genotypes B and C in the incidences of HBV DNA seroclearance, HBeAg seroclearance, HBeAg seroconversion, and ALT normalization (P > 0.05) (Figure 2).

## 5. Discussion

According to difference of sequences, HBV gene was classified into nine genotypes from A through I. HBV genotypes have a nonhomogeneous regional distribution. Genotype A and D predominate in Europe, Africa, and Mediterranean Sea, while genotype B and C predominate in Asia and North America. Genotype B predominates in south of China while Genotype C predominates in north of China (10). In this study, we used nPCR-MPP and testified that 73.6% of patients with CHB were infected by genotype B while 26.4% were infected by genotype C, which was consistent with our former report (11). Most of hepatologists believed that HBV genotype could affect antiviral efficacy of IFN-α. A large-scale multicenter study (12) on Peg-IFN showed that patients with CHB who were infected by genotypes A, B, C, or D expressed different responses to one-year treatment. Their HBeAg seroconversion rates were respectively 47%, 44%, 28%, and 25% and HBsAg seroclearance rates were respectively 14%, 9%, 3%, and 2%. Three years later, follow-up study (13) showed that their HBeAg seroconversion rates were respectively 9%, 86%, 67%, and 76% and HBsAg seroclearance rates were respectively 58%, 14%, 0%, and 6%. These data indicated that genotypes A and B had better persistent response to IFN than genotype C and D had. A recent meta-analyses (14), two retrospective studies (5, 6), and a comprehensive analysis (15) on more than 1200 patients showed that genotype A has the highest virologic response to IFN in HBeAg-positive patients, whereas genotype C has the highest virologic response to IFN in HBeAg-negative patients; hence, few HBeAg-negative patients were infected by genotype A HBV. The two retrospective studies (5, 6) also showed that HBV genotype could not affect antiviral efficacy of LAM. However, the association between HBV genotype and ADV has been always controversial. Some specialists declared that there is no association between HBV genotype and antiviral efficacy of ADV (5), but some specialists found that patients who were infected by HBV genotype D had a higher rate of resistance to ADV than those who were infected by other HBV genotypes had (16). Zeng et al. (17) studied 183 HBeAg-positive patients with CHB who received ADV treatment for one year, and found that patients infected with genotype B had a significantly higher decline in HBV DNA level (3.6 lg IU/mL) and HBV DNA seroclearance (41.8%) in comparison to those infected with genotype C (3.1 lg IU/mL, 34.6%) (P < 0.05), but this difference was not found in HBeAg-negative patients. This study indicated that perhaps HBV genotype B had a better response to ADV than genotype C had. However, Zhao et al. (18) studied 218 HBeAg-positive patients with CHB who received ADV treatment with a random, multicenter, and placebo-control method, and found that patients infected with genotypes B or C could gain significant antiviral efficacy with a dose of 10 mg/day for one year; however, there was no difference in antiviral efficacy between these two groups of patients (P > 0.05). 

ADV is the most popular antiviral drug in China, due to its low cost, good antiviral efficacy, few side effects, and convenient of administration. Therefore, it is very important to study the association between HBV genotypes and antiviral efficacy of ADV. Patients with CHB and lower HBV DNA baseline level or higher ALT baseline level will show higher rates of HBV DNA seroclearance. To eliminate disturbance from baseline level of HBV DNA or ALT, only HBsAg-positive patients with HBV DNA level of 10^4^ to 10^7^ IU/mL and ALT level of 80 to 400 U/mL were recruited. There were no significant difference in baseline level of HBV DNA and ALT between ADV group and NC group (P > 0.05), as well as between patients infected with genotypes B and C (P > 0.05). The common methods for genotyping HBV are as follows:

- restriction fragment length polymorphism (RFLP);
- gene sequencing;
- PCR microplate sandwich hybridization enzyme-linked immunoabsorbent assay;
- and line probe assay (INNO-LiPA);

(5) PCR with multiple pairs of genotype-specific primers. In this study, we used nPCR-MPP to amplify HBV DNA fragments of genotypes A through C and genotypes D through F, and to distinguish HBV genotypes only by length of PCR products, which is sensitive, repeatable, easy, and economic (19). Through detecting HBV genotypes from 526 patients with CHB, we only found genotypes B and C. Through comparing treatment efficacy after one year between ADV group and NC group, we found that patients in ADV group had gained significantly higher rates of HBV DNA seroclearance, ALT normalization, and HBeAg seroconversion in comparison with NC group (P < 0.05). The result confirmed that ADV is actually a potent antiviral drug for HBV, even though its antiviral ability is the weakest among all of nucleotide analogues. However, there were no significant differences between patients infected with genotype B and those infected with genotype C in these three rates (P > 0.05), which indicated that genotype B and C had no significant difference in virologic, biochemical, and immunologic response to ADV. Thus, for most of Chinese patients with CHB who are commonly infected with genotype B or C, effects of HBV genotypes on antiviral efficacy of ADV is not a matter of concern. Nevertheless, it is not clear whether other HBV genotypes such as A and D would affect ADV antiviral efficacy. Moreover, genotype B and C might have different variation rates, which would result in different antiviral efficacy. Further study should be conducted on these problems in the future.
